# Predicting protein complexes using a supervised learning method combined with local structural information

**DOI:** 10.1371/journal.pone.0194124

**Published:** 2018-03-19

**Authors:** Yadong Dong, Yongqi Sun, Chao Qin

**Affiliations:** Beijing Key Lab of Traffic Data Analysis and Mining, School of Computer and Information Technology, Beijing Jiaotong University, Beijing, China; Koç University, TURKEY

## Abstract

The existing protein complex detection methods can be broadly divided into two categories: unsupervised and supervised learning methods. Most of the unsupervised learning methods assume that protein complexes are in dense regions of protein-protein interaction (PPI) networks even though many true complexes are not dense subgraphs. Supervised learning methods utilize the informative properties of known complexes; they often extract features from existing complexes and then use the features to train a classification model. The trained model is used to guide the search process for new complexes. However, insufficient extracted features, noise in the PPI data and the incompleteness of complex data make the classification model imprecise. Consequently, the classification model is not sufficient for guiding the detection of complexes. Therefore, we propose a new robust score function that combines the classification model with local structural information. Based on the score function, we provide a search method that works both forwards and backwards. The results from experiments on six benchmark PPI datasets and three protein complex datasets show that our approach can achieve better performance compared with the state-of-the-art supervised, semi-supervised and unsupervised methods for protein complex detection, occasionally significantly outperforming such methods.

## Introduction

A group of proteins that interact with one another for specific biological activities is called a protein complex [1]. Predicting protein complexes is helpful for understanding the principles of cellular tissue [2, 3], predicting protein functions [4], identifying disease genes [5] and discovering drug-disease associations [6]. Modern experimental techniques have revealed a large amount of protein interactions, thereby enabling protein complexes to be predicted from protein-protein interaction (PPI) networks.

In recent years, automatic computational approaches have increasingly been proposed for detecting protein complexes from PPI networks [7]. A PPI network can be represented as an undirected graph, where the nodes denote the proteins and the edges denote the interactions [8]. Existing protein complex detection approaches can be broadly grouped into two categories: unsupervised and supervised learning methods. The majority of the unsupervised methods detect protein complexes by discovering the densely connected subgraphs in the PPI network using predefined rules. The Markov clustering method (MCL) partitions the PPI network into densely connected subgraphs by simulating random walks within the graph [9]. The molecular complex detection (MCODE) method isolates the dense regions by growing the local weighted seeds [10]. The restricted neighbourhood search clustering (RNSC) method partitions networks into clusters based on a cost function, which is assigned to each partitioning [11]. The CFinder method discovers clusters by combining adjacent *k*-cliques identified via clique percolation [12, 13]. The clustering based on maximal cliques (CMC) [2] method is also a clique-based method that detects complexes by removing or merging cliques based on their inter-connectivity. The repeated random walks (RRW) [14] method implicitly utilizes network topology, edge weights and long-range interactions by repeated random walks to identify protein complexes. The clustering with overlapping neighbourhood expansion (ClusterONE) [15] method finds subgraphs with high cohesiveness by greedy adding or removing nodes starting from seed nodes.

**Fig 1:**
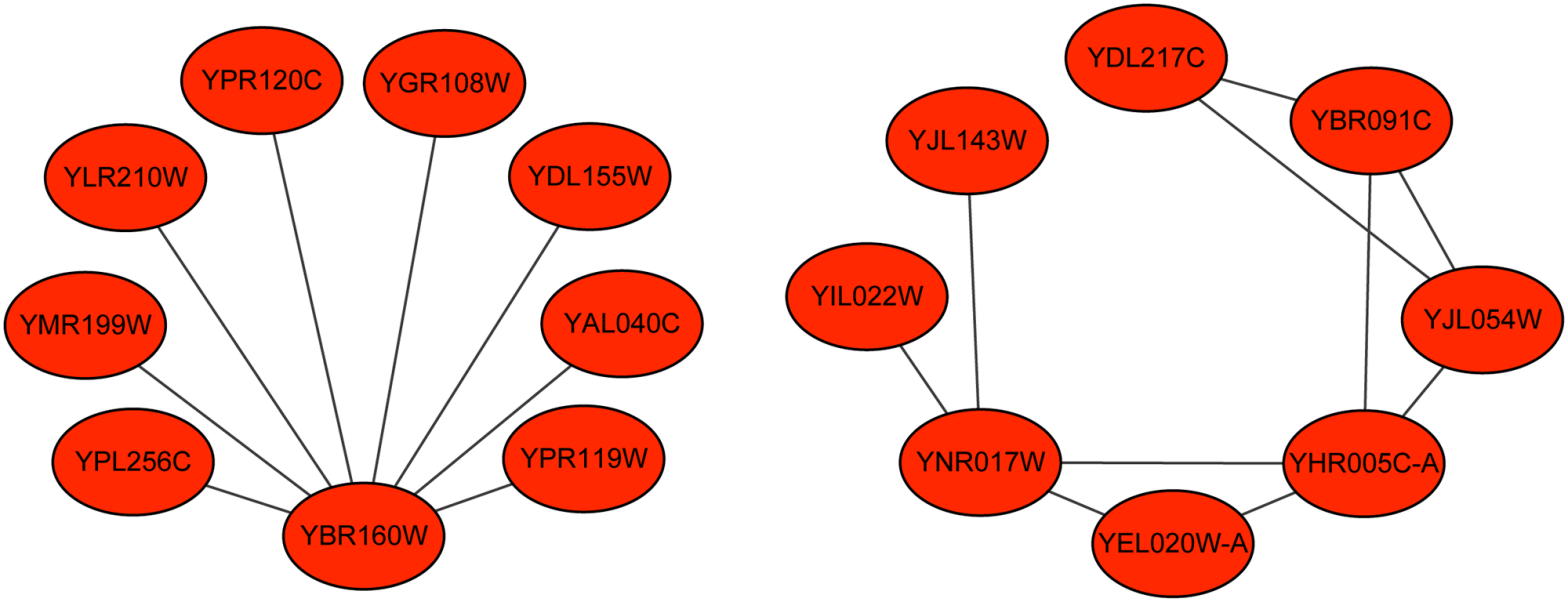
Two sparse complexes in the MIPS complex catalogue database.

The majority of the unsupervised methods are simply based on the topological structure of the PPI network and do not utilize the information of the existing true complexes [7]. These methods assume that protein complexes are in dense regions of PPI networks, but in fact, many true complexes are in sparse regions. Fig 1 shows two sparse complexes from the Munich Information Center for Protein Sequences (MIPS) complex catalogue database [16]. Therefore, using topological attributes alone is not sufficient for efficiently detecting protein complexes [8].

In recent years, supervised learning methods have been developed to detect complexes by utilizing the informative properties of known complexes. These types of methods consist of three main steps: 1) extract useful features from the known complexes and denote them as vectors, 2) train a supervised classification model or score function to distinguish the true protein complexes from random subgraphs based on the extracted features, and 3) search for protein complexes from PPI networks using the trained classification model or score function as a guide. For example, SCI-BN [17] is a supervised method that trains a probabilistic Bayesian network to score the subgraphs. RM [7] trains a regression model to score the subgraphs. NN [18] is a semi-supervised method, and it trains a neural network model on the training sets and uses the trained model to detect new protein complexes. Then, NN uses the new predicted complexes to adjust the parameters of the model recursively until the model converges. The final converged neural network model is used to guide the search process for detecting protein complexes. ClusterEPs [8] defines an integrative score of emerging patterns (EPs) to measure the likelihood of a subgraph being a complex.

The supervised methods extract features from true complexes and learn a prediction model, and then they use the model as a guide in the protein complex search process. However, the PPI data contain considerable amounts of noise, and many of the benchmark clusters are incomplete; thus, the trained prediction model is inaccurate. The existing supervised methods only use the prediction model to guide the search process for detecting protein complexes. In this paper, we first define a new score function that combines a supervised model with unsupervised structural information. Based on this score function, we propose a search algorithm that works both forwards and backwards to identify protein complexes from PPI networks. We use a neural network as the classification model, and we adjust the output of the neural network at each step using the local structural information. Our method is named ClusterSS (clustering with supervised and structural information).

To assess the performance of ClusterSS, we compared ClusterSS with supervised, semi-supervised and unsupervised learning methods. First, we compared ClusterSS with three supervised learning methods, namely, ClusterEPs [8], SCI-BN [17] and RM [7], and with the semi-supervised learning method NN [18]. The results showed that ClusterSS achieved considerably better performance (precision, recall and F1) on the commonly used DIP PPI network [19]. We then compared ClusterSS with seven unsupervised learning methods: MCL [9], MCODE [10], RNSC [11], CFinder [12, 13], CMC [2], RRW [14] and ClusterONE [15]. The PPI datasets are five large-scale yeast PPIs, including Collins, Krogan core, Krogan extended, Gavin and BioGRID. The two protein complex datasets are the MIPS complex catalogue database [16] and the Saccharomyces Genome Database (SGD) [20]. Comparative experiments showed that ClusterSS achieved the highest fraction score and maximum matching ratio (MMR) score among all seven literature methods on all five PPI datasets and a higher composite score than the other methods.

In case studies, we analyzed the prediction results of ClusterSS, ClusterEPs and ClusterONE on five protein complexes. The results indicated that only ClusterSS could detect the origin recognition complex (ORC) and the Pwp2p-containing subcomplex of 90S preribosome complex completely and correctly. From the gene ontology (GO) analysis, we obtained four predicted clusters that have not previously been identified as complexes. However, their low p-values suggest that these clusters are very likely complexes in the biological sense.

The algorithm has been implemented in Python, and both the software and source code are available from the authors.

## Methods

A PPI network can be represented as an undirected graph *G* = (*V*, *E*, *W*), where *V* denotes the set of nodes (proteins), *E* denotes the set of edges (interactions), and *W* denotes the weights of the edges. Let *S* = (*V*_*S*_, *E*_*S*_, *W*_*S*_) be a subgraph of *G*, and let *N*_*ext*_(*G*, *S*) be the external neighbours of *S* of *G*, which is defined as follows:

$$\begin{array}{c} N_{ext}\left(G,S\right)=\left\{v|\left(w,v\right)\in E,v\in V-V_{S},w\in V_{S}\right\}. \end{array}$$ (1)

**Fig 2:**
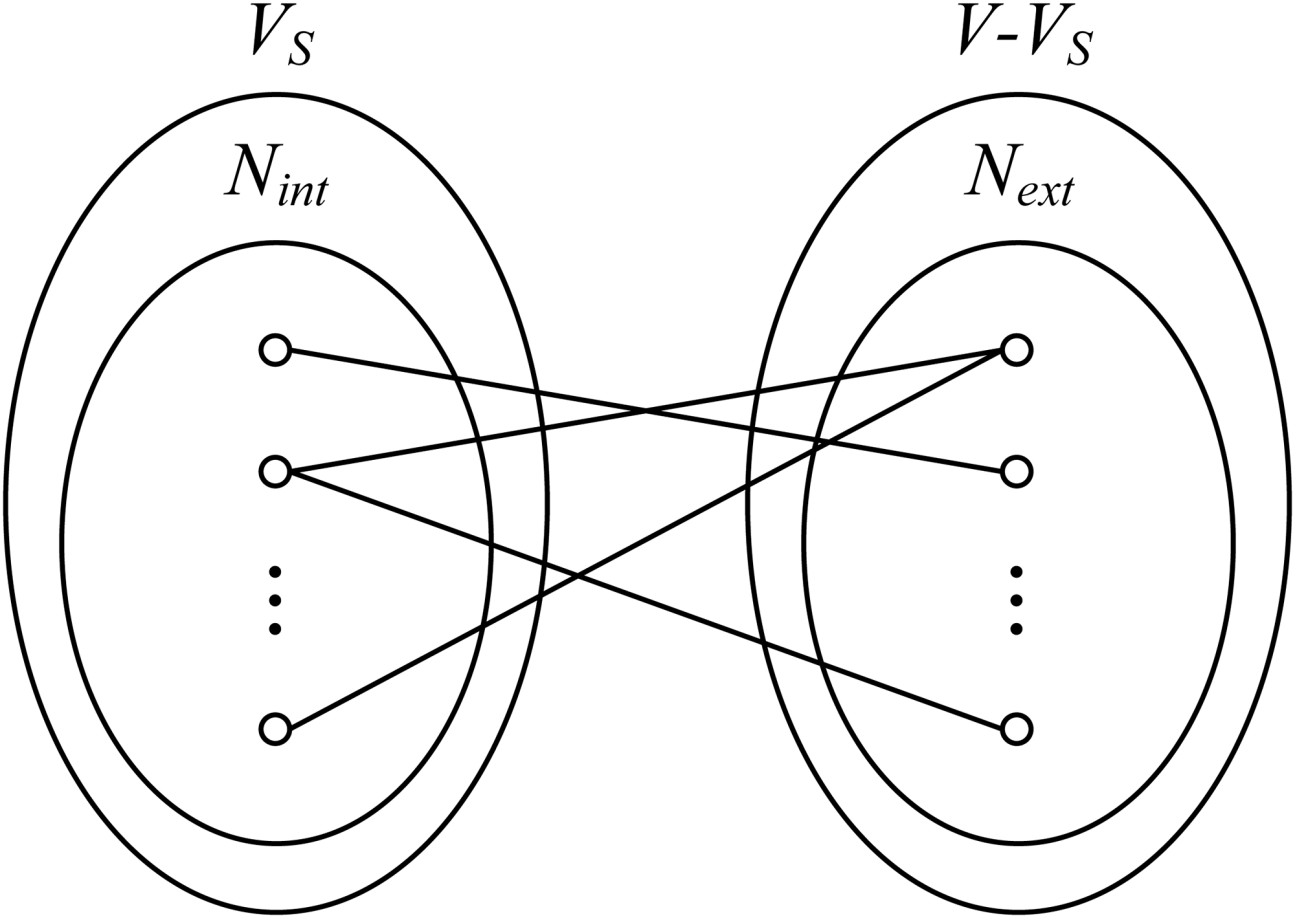
Illustrations of *N*_*ext*_ and *N*_*int*_.

Opposite to *N*_*ext*_(*G*, *S*), we define *N*_*int*_(*G*, *S*) as follows:

$$\begin{array}{c} N_{int}\left(G,S\right)=\left\{v|\left(w,v\right)\in E,v\in V_{S},w\in V-V_{S}\right\}, \end{array}$$ (2)

that is, the nodes in *N*_*int*_(*G*, *S*) have edges with the nodes in *V* − *V*_*S*_. Illustrations of *N*_*ext*_(*G*, *S*) and *N*_*int*_(*G*, *S*) are shown in Fig 2.

Our ClusterSS method includes three main steps: 1) extracting features, 2) determining the score function, and 3) searching for complexes in PPI networks. These steps are described in detail in the following subsections.

### Extracting features

To measure the similarity between complexes, we represent each subgraph as a feature vector. First, we select 24 features, which are divided into 9 groups, as follows: 1) node size (the number of nodes in subgraph *S*), 2) graph density (the density of subgraph *S*), 3) degree statistics, 4) edge weight, 5) degree correlation statistics, 6) clustering coefficient statistics, 7) topological coefficients, 8) first eigenvalues, and 9) protein weight/size statistics (see S1 Table for details). Second, by performing a sequential backward feature selection, we remove two feature groups: degree correlation and protein weight/size. Thus, the feature vector contains 7 groups, which include 17 features. Finally, we extract all 17 features from the subgraphs, mapping from the true complexes in the training set, and we denote them as the positive instances. For each true complex in the training set, we produce 20 complex-unlikely random subgraphs with the same size, and we extract features from these subgraphs as the negative instances. Therefore, the negative instances are 20 times the number of positive instances and obey the same distribution. We place the positive and negative instances together and denote it as dataset *D* for training the supervised neural network model.

### Determining the score function

**Fig 3:**
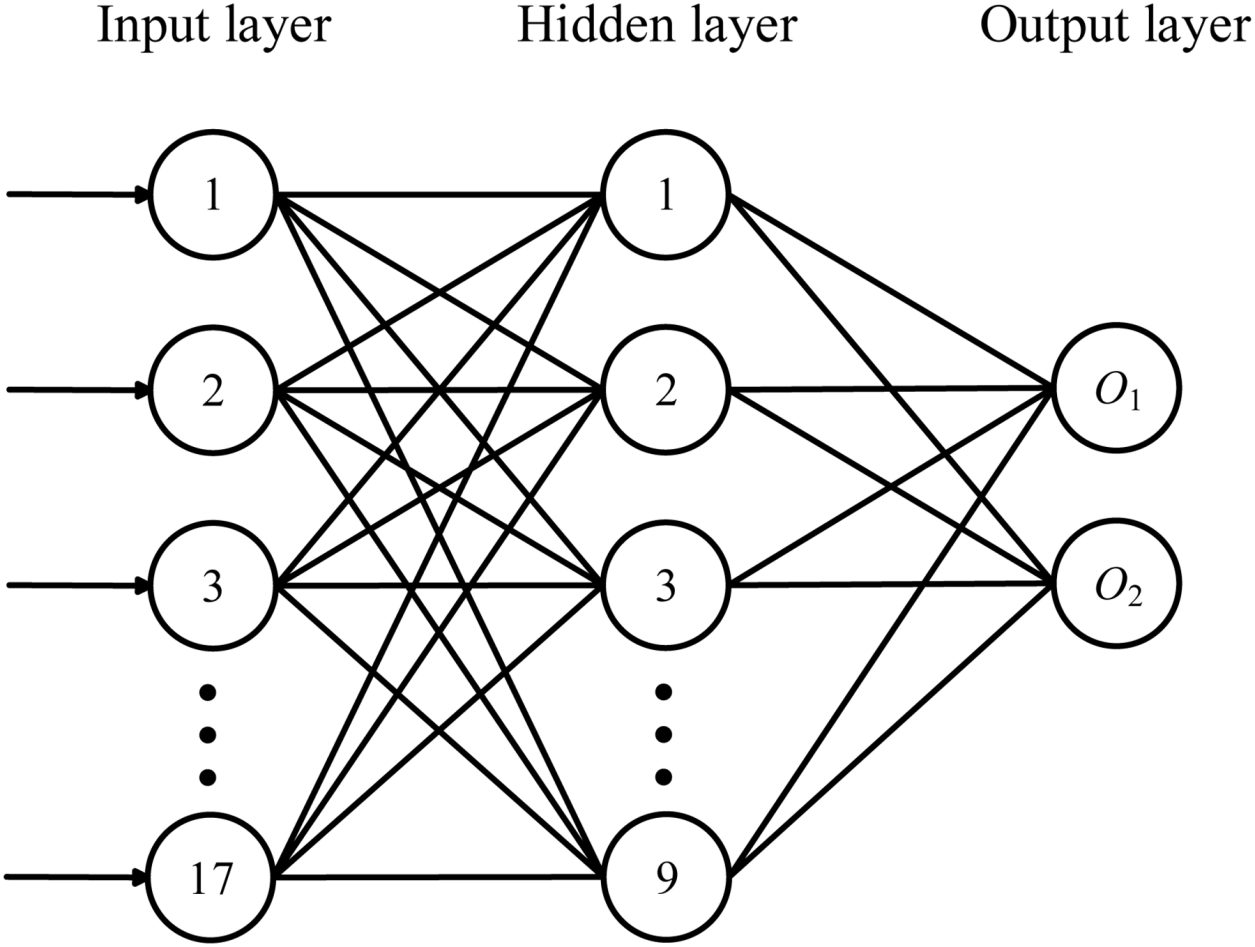
The structure of the neural network.

For dataset *D* derived from the input PPI network, a neural network model is trained to fit the probability of subgraph *S* belonging to true complexes (as shown in Fig 3). We choose a three-layer fully connected neural network. The input layer contains 17 nodes according to the number of features in *D*, the hidden layer contains 9 nodes, and the output layer contains two nodes, *O*_1_ and *O*_2_ (the details of the parameters are shown in S1 Text). Given a subgraph *S* of *G*, we can calculate the two outputs *O*_1_(*G*, *S*) and *O*_2_(*G*, *S*). By normalizing *O*_1_(*G*, *S*), we obtain the supervised score,

$$\begin{array}{c} supervisedScore\left(G,S\right)=\frac{O_{1}\left(G,S\right)}{O_{1}\left(G,S\right)+O_{2}\left(G,S\right)}, \end{array}$$ (3)

where *O*_1_(*G*, *S*) denotes the probability of subgraph *S* belonging to true complexes and *O*_2_(*G*, *S*) denotes the probability of subgraph *S* belonging to false complexes. The higher the supervised score is for subgraph *S*, the higher is the probability that it belongs to true complexes.

To improve the supervised model, we introduce the structural score, which was used by ClusterONE [15] to make the score function more robust and accurate. The structural score is defined as follows:

$$\begin{array}{c} structuralScore\left(G,S\right)=\frac{w_{in}\left(G,S\right)}{w_{in}\left(G,S\right)+w_{out}\left(G,S\right)}, \end{array}$$ (4)

where *w*_*in*_(*G*, *S*) denotes the total weight of edges of subgraph *S* of *G* and *w*_*out*_(*G*, *S*) denotes the total weight of edges between node sets *V*_*s*_ and *V* − *V*_*s*_ in *G*. The higher the structural score is for subgraph *S*, the higher is the probability that it belongs to true complexes. The final clustering score *f*(*G*, *S*) used to guide the search process is defined as follows:

$$\begin{array}{c} f\left(G,S\right)=\frac{supervisedScore(G,S)+structuralScore(G,S)}{2} \end{array}$$ (5)

The values of *f*(*G*, *S*) range from 0 to 1. A larger *f*(*G*, *S*) suggests that *S* is more likely to be a protein complex.

### Searching for new complexes

The clustering score *f*(*G*, *S*) is used as a heuristic function in the process of searching for new complexes in graph *G*. First, we need to determine the start nodes of the search process, and we consider them to be the initial clusters. Then, these clusters are updated according to the heuristic function *f*(*G*, *S*).

We choose a both forwards and backwards strategy in the complex search process. At each search step, the current candidate cluster is denoted as *C*. We go through all the nodes in *N*_*ext*_(*G*, *C*) to find a node *u* that maximizes the score function *f*(*G*, *C*∪{*u*}) and go through all the nodes in *N*_*int*_(*G*, *C*) to find a node *v* that maximizes the score function *f*(*G*, *C* − {*v*}). Then, *u* is added to *C* if *f*(*G*, *C*∪{*u*}) is higher than *f*(*G*, *C* − {*v*}), and *v* is deleted from *C* otherwise. The asymptotic time complexity of this process is approximately *O*(*n*^3^); this process is very time consuming, particularly when the scale of *G* is relatively large. Thus, we design a trick called top-*k* to accelerate the search process. Let *BS* be the bipartite subgraph of *G* induced by node sets *N*_*ext*_(*G*, *C*) (see Eq 1) and *N*_*int*_(*G*, *C*) (see Eq 2), as shown in Fig 2. We sort the nodes of *N*_*ext*_(*G*, *C*) and *N*_*int*_(*G*, *C*) in descending order according to their degrees in *BS*, and we take the first *k* nodes in each of the two sets as the candidate node sets, denoted as $N_{ext}^{k}\left(G,C\right)$ and $N_{int}^{k}\left(G,C\right)$, respectively. At each search step, the candidate nodes are selected from $N_{ext}^{k}\left(G,C\right)$ (or $N_{int}^{k}\left(G,C\right)$) rather than *N*_*ext*_(*G*, *C*) (or *N*_*int*_(*G*, *C*)); thus, the search process becomes quicker. In fact, we take *k* = 5 in our experiments (S6 Table presents the running time comparison of our top-*k* trick). We also design a hyper-parameter *α* to control the growth scale of candidate complexes. The search process will stop when the new score function is less than *α* times the old function. A larger value of *α* will cause the search process to complete earlier. We set the value of *α* to 1.02 in all the experiments (S2 and S3 Tables present the performance comparison of ClusterSS with different values of *α*). The details of the algorithm are shown in Algorithm 1.

The final step is to merge the highly overlapping clusters as in [15]. We also merge each pair of cluster with an overlapping score *ω* [10] that is no less than the threshold of 0.9. The overlapping score of two clusters *A* and *B* is defined as follows:

$$\begin{array}{c} \omega \left(A,B\right)=\frac{{\left|A\cap B\right|}^{2}}{\left|A|\times |B\right|}. \end{array}$$ (6)

If we find two clusters in which their overlapping score is not less than the threshold, we merge them and add them to the protein complex candidates. This process is performed iteratively until there is no pair of clusters with an overlapping score that satisfies the threshold.

**Algorithm** 1 **The algorithm of ClusterSS**

**Input**: *G*: the PPI network; *T*: the training set containing known complexes; *α*: a hyper-parameter that controls the growth scale of candidate complexes;

**Output**: *P*: the set of predicted complexes;

1: **for** each *cluster* ∈ *T* **do**

2:  Extract 17 features from *cluster* and treat them as positive instances and add them to instance set *D*;

3:  Generate 20 subgraphs of *G* randomly with the same size as *cluster*; extract 17 features from each of them and treat them as negative instances and add them to instance set *D*;

4: **end for**

5: Train a neural network model on dataset *D*; then, determine the supervised score function based on Eq 3, and then obtain the adjusted score function *f*(*G*, *S*) based on Eq 5;

6: Find the nodes in *G* with a degree of greater than 1 as the start nodes and denote it as *ST*;

7: **for** each *v*_0_ ∈ *ST* **do**

8:  Initialize candidate cluster *C* = {*v*_0_}, and calculate the score function *f*(*G*, *C*);

9:  **repeat**

10:   $u=\arg\;\max_{u\in N_{ext}^{k}\left(G,C\right)}f\left(G,C\cup \left\{u\right\}\right)$;

11:   $v=\arg\;\max_{v\in N_{int}^{k}\left(G,C\right)}f\left(G,C-\left\{v\right\}\right)$;

12:   **if** *f*(*G*, *C* ∪ {*u*}) ≥ *f*(*G*, *C* − {*v*}) **then**

13:    update *C*′ = *C* ∪ {*u*};

14:   **else**

15:    update *C*′ = *C* − {*v*};

16:   **end if**

17:   **if** *f*(*G*, *C*′) > *αf*(*G*, *C*) **then**

18:    update *C* = *C*′;

19:   **end if**

20:  **until** (*f*(*G*, *C*′) ≤ *αf*(*G*, *C*))

21:  Add candidate cluster *C* to set *P*;

22: **end for**

23: **for** each pair of clusters *c*_*i*_ and *c*_*j*_ in *P* **do**

24:  **if** *w*(*c*_*i*_, *c*_*j*_) > 0.9 according to Eq 6 **then**

25:   Merge *c*_*i*_ and *c*_*j*_ and add it to *P*;

26:  **end if**

27: **end for**

28: **return** *P*

## Results and discussion

This section consists of four parts. We first compare the performance of ClusterSS with those of supervised and semi-supervised methods. Then, we present our results of the comparison with the unsupervised methods. In the third part, we analyze two examples of detected protein complexes. Finally, we present the GO analysis on the novel protein complexes predicted by our method.

### Evaluation measures and datasets

We used six PPI datasets and three benchmark protein complex datasets in all the experiments. The PPI datasets include the DIP dataset [19], the Gavin dataset [21], the Krogan core dataset [22], the Krogan extended dataset [22], the Collins dataset [23] and the BioGRID dataset [24].

The detailed properties of the PPI datasets are shown in Table 1. The benchmark protein complex datasets include the TAP06 [21] dataset, the MIPS dataset [16] and the SGD [20] dataset.

**Table 1:** Properties of the protein-protein interaction datasets.

dataset	protein	interactions	reference
DIP	4931	22277	Xenarios et al. [19]
Gavin	1855	7669	Gavin et al. [21]
Krogan core	2708	7123	Krogan et al. [22]
Krogan extended	3672	14317	Krogan et al. [22]
Collins	1622	9074	Collins et al. [23]
BioGRID	5640	59748	Stark et al. [24]

Similar to ClusterEPs, we use three measures, namely, precision, recall and F1-measure, to evaluate the performance of the supervised learning methods. Recall measures the ratio of complexes in the benchmark datasets that match at least one complex in the predicted protein complex datasets, and precision measures the ratio of complexes in the predicted protein complex datasets that match at least one of the complexes in the benchmark datasets. F1-measure is the harmonic mean of precision and recall. Let *B* = {*b*_1_, *b*_2_, ⋯, *b*_*i*_, ⋯, *b*_*m*_} denote the benchmark complex datasets, and let *P* = {*p*_1_, *p*_2_, ⋯, *p*_*j*_, ⋯, *p*_*n*_} denote the protein complex sets predicted by a method, where *b*_*i*_ and *p*_*j*_ represent the *i*^*th*^ and *j*^*th*^ complexes in *B* and *P*, respectively, and *m* and *n* represent the number of complexes in *B* and *P*, respectively. For two protein complexes *b*_*i*_ and *p*_*j*_, if the overlapping score *ω*(*b*_*i*_, *p*_*j*_) [25] as defined in Eq 6 is greater than or equal to 0.25, then *b*_*i*_ and *p*_*j*_ are considered to be matching. Let *N*_*bp*_ be the number of the benchmark complexes that match at least one predicted complex, and let *N*_*pb*_ be the number of the predicted complexes that match at least one of the benchmark complexes; then, *N*_*bp*_ and *N*_*pb*_ are defined as follows:

$$\begin{array}{c} N_{bp}=\left|\left\{b|b\in B,\exists p\in P,\omega \left(b,p\right)\geq 0.25\right\}\right|, \end{array}$$ (7)

$$\begin{array}{c} N_{pb}=\left|\left\{p|p\in P,\exists b\in B,\omega \left(b,p\right)\geq 0.25\right\}\right|. \end{array}$$ (8)

The precision, recall and F1-measure are defined as follows:

$$\begin{array}{c} precision=\frac{N_{pb}}{n}, \end{array}$$ (9)

$$\begin{array}{c} recall=\frac{N_{bp}}{m}, \end{array}$$ (10)

$$\begin{array}{c} F1-measure=\frac{2\times precision\times recall}{precision+recall}. \end{array}$$ (11)

To compare with unsupervised methods, we use three measures: fraction (Frac), geometric accuracy (ACC) and MMR [8]. The definition of fraction is the same as that of recall. ACC is the geometric mean of clustering-wise sensitivity (Sn) and clustering-wise positive predictive value (PPV) [3]. Let *T* be an *n* × *m* matrix, and let *T*_*ij*_ represent the number of proteins found in both *b*_*i*_ and *p*_*j*_. Then, *Sn*(*B*, *P*), *PPV*(*B*, *P*) and *ACC*(*B*, *P*) are calculated as follows:

$$\begin{array}{c} Sn\left(B,P\right)=\frac{\sum_{i=1}^{m}\max_{j=1}^{n}T_{ij}}{\sum_{i=1}^{m}\left|b_{i}\right|}, \end{array}$$ (12)

$$\begin{array}{c} PPV\left(B,P\right)=\frac{\sum_{j=1}^{n}\max_{i=1}^{m}T_{ij}}{\sum_{j=1}^{n}\sum_{i=1}^{m}T_{ij}}, \end{array}$$ (13)

$$\begin{array}{c} Acc\left(B,P\right)=\sqrt{Sn(B,P)\times PPV(B,P)}, \end{array}$$ (14)

where |*b*_*i*_| represents the number of proteins in complex *b*_*i*_.

The MMR [15] is a measure that is based on the maximal one-to-one mapping between *B* and *P*, and it explicitly penalizes cases where a benchmark complex is split into two or more parts in the predicted set because only one part is allowed to match the benchmark complexes [15]. The MMR is calculated as follows: 1) construct a bipartite graph *BG* between *B* and *P*, in which each cluster is represented as a node; 2) for each cluster *b*_*i*_ in *B* and each cluster *p*_*j*_ in *P*, connect *b*_*i*_ and *p*_*j*_ by an edge with a weight of *ω*(*b*_*i*_, *p*_*j*_) if *ω*(*b*_*i*_, *p*_*j*_) > 0; 3) select disjoint edges from *BG* to maximize the sum of their weights; and 4) the MMR is the total weights of the selected edges divided by |*B*|.

### Comparison with supervised and semi-supervised learning methods

In this part, we first compare the prediction performance of ClusterSS with three existing supervised methods, namely, SCI-BN [17], RM [7] and ClusterEPs [8], and with the semi-supervised method NN [18] on the DIP [19] dataset, which follows the approach used by ClusterEPs. Considering that ClusterEPs is the most recent supervised method, we subsequently compared it with ClusterSS in detail on the other five datasets, including the Gavin dataset [21], the Krogan core dataset [22], the Krogan extended dataset [22], the Collins dataset [23] and the BioGRID dataset [24].

Because the programs of SCI-BN and RM are not available, ClusterEPs compared them based on their published results; therefore, we also compared with their published results. The PPI dataset for the test is the DIP [19] dataset. SCI-BN used an SVM-based method to filter out the interactions that have a score below 1.0. RM used a GO-based method to filter out the interactions that have a GO score of less than 0.9. ClusterEPs preprocessed the PPI network using the topological clustering semantic similarity (TCSS) [26] method and filtered out the interactions that have a biological process (BP) score of less than 0.5. ClusterSS employed the same processing method as ClusterEPs.

The true protein complex datasets for the test are the two independent datasets MIPS [16] and TAP06 [21]. We removed the complexes composed of a single or pair of proteins from the two datasets. There are 195 complexes remaining in MIPS and 193 complexes remaining in TAP06 after preprocessing. There are a total of 1579 proteins in the MIPS and TAP06 complex datasets, and we extracted a PPI subgraph of these proteins from DIP. Then, we tested ClusterSS on this PPI graph.

To assess the protein complex identification performance, we performed the experiments using MIPS as the positive training set and TAP06 as the test set and vice versa. We chose three measures, namely, precision, recall and F1, to evaluate the performance. The results are presented in Table 2. As shown in this table, when MIPS was considered as the training set and TAP06 as the test set, ClusterSS achieved the highest scores on all three measures. Specifically, the F1 measure of ClusterSS was 12.0 percentage points higher than that of ClusterEPs, 61.2 percentage points higher than that of SCI-BN and 43.1 percentage points higher than that of RM. When TAP06 was used as the training set and MIPS as the test set, the F1 measure of ClusterSS was slightly higher than that of ClusterEPs. ClusterEPs has a higher precision score; however, ClusterSS has a considerably higher recall score. Both of these methods have higher scores compared with SCI-BN and RM on all three measures.

**Table 2:** Performance compared with ClusterEPs, SCI-BN and RM on the DIP dataset.

Train	Test	Method	Precision	Recall	F1
MIPS	TAP	ClusterSS	**0.477**	**0.864**	**0.614**
MIPS	TAP	ClusterEPs	0.424	0.782	0.548
MIPS	TAP	SCI-BN	0.312	0.489	0.381
MIPS	TAP	SCI-SVM	0.247	0.377	0.298
MIPS	TAP	RM	0.424	0.433	0.429
TAP	MIPS	ClusterSS	0.526	**0.807**	**0.636**
TAP	MIPS	ClusterEPs	**0.606**	0.664	0.633
TAP	MIPS	SCI-BN	0.219	0.537	0.312
TAP	MIPS	SCI-SVM	0.176	0.379	0.240
TAP	MIPS	RM	0.489	0.525	0.506

As a semi-supervised learning model, NN [18] was evaluated using MIPS as both the training set and test set; thus, we tested ClusterSS under the same settings. The results are presented in Table 3. As shown in this table, ClusterSS has considerably higher scores compared with NN and other supervised methods on all three measures. Specifically, the F1 measure of ClusterSS was 90.4 percentage points higher than that of NN, 8.8 percentage points higher than that of ClusterEPs, and substantially better than those of SCI-BN and RM.

**Table 3:** Performance compared with NN on the DIP dataset.

Train	Test	Method	Precision	Recall	F1
MIPS	MIPS	ClusterSS	**0.690**	**0.836**	**0.756**
MIPS	MIPS	ClusterEPs	0.649	0.751	0.695
MIPS	MIPS	SCI-BN	0.273	0.473	0.346
MIPS	MIPS	SCI-SVM	0.239	0.412	0.302
MIPS	MIPS	RM	0.419	0.670	0.514
MIPS	MIPS	NN	0.333	0.491	0.397

In the following, we conducted further comparisons between ClusterEPs and ClusterSS on the other five PPI datasets. ClusterEPs trained models on the training sets and then searched for complexes on the subgraphs of PPI networks. The subgraphs only consist of those proteins that exist in the training set or in the test set. We compared the performances of ClusterSS and ClusterEPs under the same conditions and same measures for a fair comparison. The measures include fraction, accuracy and MMR, and the sum of these three measures is denoted as the composite score. Because ClusterSS and ClusterEPs need negative instances selected randomly in the training process, we ran ClusterEPs and ClusterSS 20 times to calculate the average performance. We first conducted the experiment using MIPS as the positive training set and using SGD as the test set. The results are presented in Table 4. As shown in this table, ClusterSS outperformed ClusterEPs on all five datasets. We then conducted the experiment using SGD as the positive training set and using MIPS as the test set. The results are presented in Table 5. As shown in this table, ClusterSS achieved a higher fraction score, accuracy score and composite score than ClusterEPs on all five datasets. Except for Gavin, ClusterSS achieved a higher MMR score on the other four datasets.

**Table 4:** Performance compared with ClusterEPs on four yeast PPI datasets using SGD as the test set.

Dataset	Method	#cluster	Frac	Acc	MMR	Composite score
Collins	ClusterSS	259	**0.876**	**0.711**	**0.603**	**2.190**
	ClusterEPs	173	0.720	0.628	0.470	1.819
Krogan core	ClusterSS	261	**0.812**	**0.639**	**0.557**	**2.007**
	ClusterEPs	291	0.626	0.574	0.422	1.622
Krogan extended	ClusterSS	280	**0.725**	**0.613**	**0.484**	**1.822**
	ClusterEPs	516	0.631	0.540	0.394	1.565
Gavin	ClusterSS	168	**0.820**	**0.684**	**0.516**	**2.020**
	ClusterEPs	255	0.803	0.635	0.500	1.939
BioGRID	ClusterSS	1060	**0.721**	**0.562**	**0.482**	**1.766**
	ClusterEPs	817	0.664	0.522	0.392	1.579

**Table 5:** Performance compared with ClusterEPs on four yeast PPI datasets using MIPS as the test set.

Dataset	Method	#cluster	Frac	Acc	MMR	Composite score
Collins	ClusterSS	293	**0.802**	**0.508**	**0.445**	**1.754**
	ClusterEPs	173	0.667	0.506	0.395	1.567
Krogan core	ClusterSS	245	**0.773**	**0.448**	**0.418**	**1.639**
	ClusterEPs	371	0.620	0.402	0.336	1.358
Krogan extended	ClusterSS	269	**0.686**	**0.423**	**0.373**	**1.482**
	ClusterEPs	516	0.585	0.383	0.301	1.268
Gavin	ClusterSS	167	**0.723**	**0.479**	0.384	**1.585**
	ClusterEPs	242	0.697	0.454	**0.390**	1.542
BioGRID	ClusterSS	984	**0.648**	**0.378**	**0.370**	**1.396**
	ClusterEPs	901	0.608	0.356	0.287	1.251

### Comparison with unsupervised learning methods

In this part, we compare the performance of ClusterSS with seven representative unsupervised approaches: MCL [9], MCODE [10], RNSC [11], CFinder [12, 13], CMC [2], RRW [14] and ClusterONE [15]. The experiment was conducted on five large-scale yeast PPI networks, including the Gavin dataset [21], the Krogan core dataset [22], the Krogan extended dataset [22], the Collins dataset [23] and the BioGRID dataset [24]. The benchmark complex sets are the MIPS dataset [16] and the SGD dataset [20]. The three evaluation measures are the Frac, the Acc and the MMR, and we denote the sum of the three measures as the composite score. For a fair comparison, all parameters of the other seven methods on every PPI dataset were the same as those used in ClusterONE. To compare with the unsupervised methods, ClusterSS searched for complexes on the entire PPI networks rather than their subgraphs.

**Fig 4:**
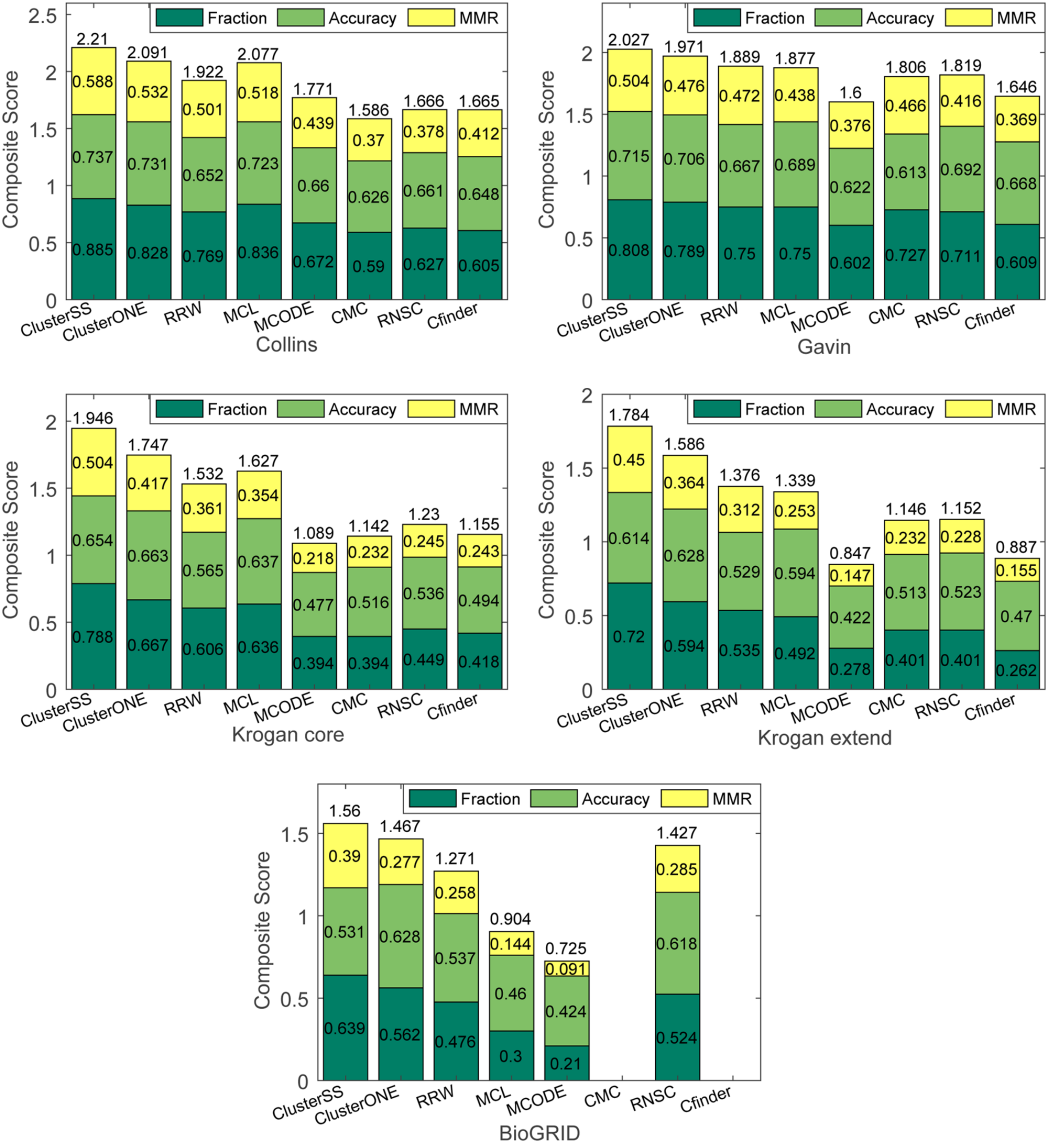
Performance comparison of eight algorithms on four yeast PPI datasets using SGD as the test set.

**Fig 5:**
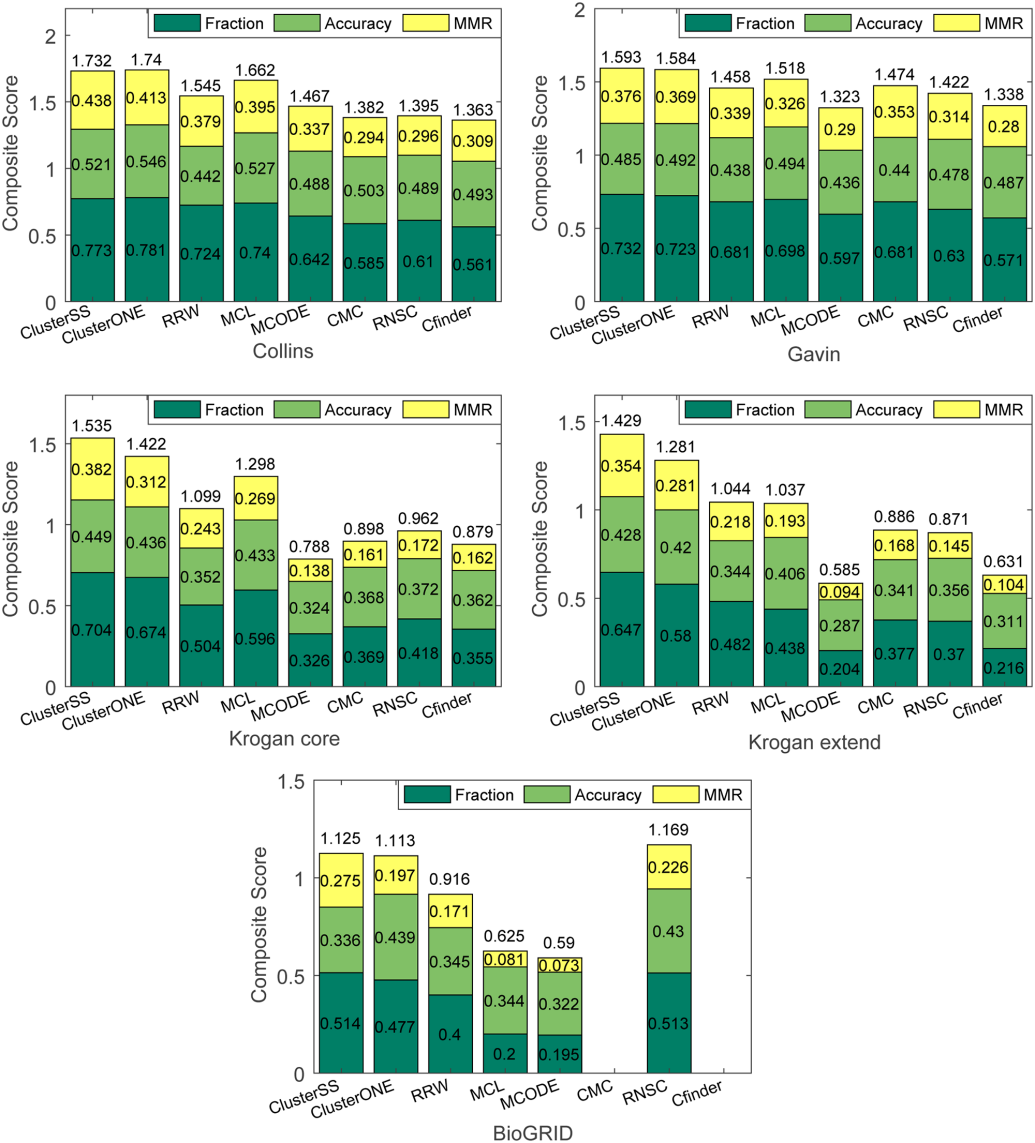
Performance comparison of eight algorithms on four yeast PPI datasets using MIPS as the test set.

We first conducted the experiment using MIPS as the positive training set and using SGD as the test set. The results are presented in Fig 4. As shown in this figure, ClusterSS achieved the highest fraction, MMR and composite score on all five PPI datasets. We then conducted the experiment using SGD as the positive training set and using MIPS as the test set. The results are presented in Fig 5. As shown in this figure, ClusterSS achieved the highest fraction score and MMR score on all five datasets. ClusterSS did not achieve the highest accuracy and composite score on the Collins and Gavin datasets, but the scores are close to the highest score and are significantly higher than those of the other six methods. We do not provide the results of CFinder and CMC on the BioGRID dataset because CFinder did not provide any results within 24 hours and CMC predicted an exorbitantly large number of clusters (more than 6000) [8]. In addition, the composite scores of ClusterSS in Figs 4 and 5 are slightly lower than those in Tables 4 and 5. The main reason for this result is that ClusterSS searched for complexes on the entire PPI networks in this section, whereas it searched for complexes on the subgraphs in the previous section.

### Case study

ClusterEPs and ClusterONE are the latest supervised and unsupervised protein complex detection methods; thus, we present a detailed case study of ClusterSS, ClusterEPs and ClusterONE on three non-overlapping complexes and a pair of overlapping complexes. The three non-overlapping complexes include the retromer complex, the Pwp2p-containing subcomplex of 90S preribosome and the DASH complex. The pair of non-overlapping complexes are the RSC and the SWI/SWF complexes.

**Fig 6:**
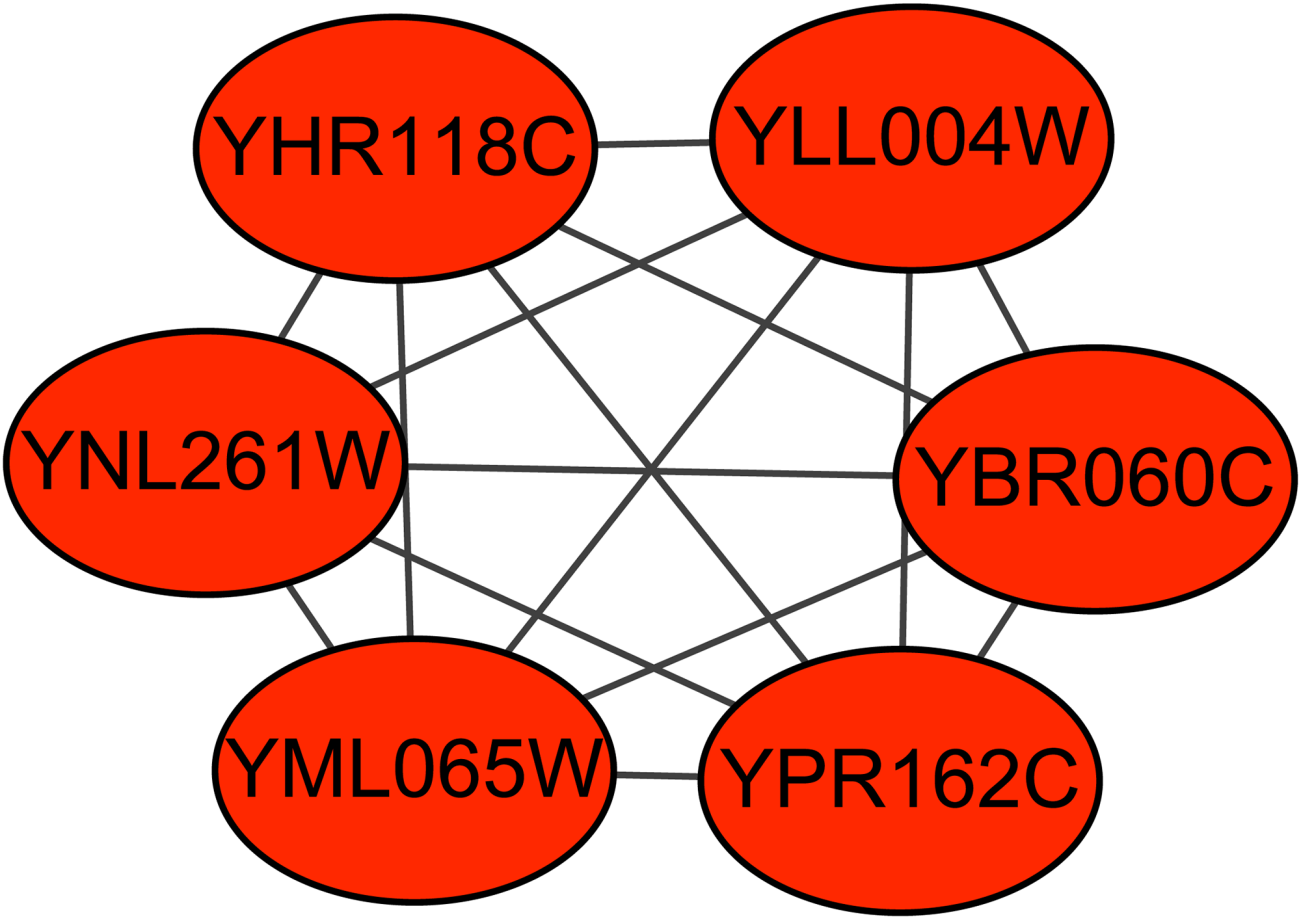
The retromer complex predicted by ClusterSS. The red nodes represent the proteins in the true complex that are detected by the algorithm, the green nodes represent the proteins in the true complex that are not detected by the algorithm, and the blue nodes represent the proteins that do not belong to the true complex that are detected by the algorithm.

**Fig 7:**
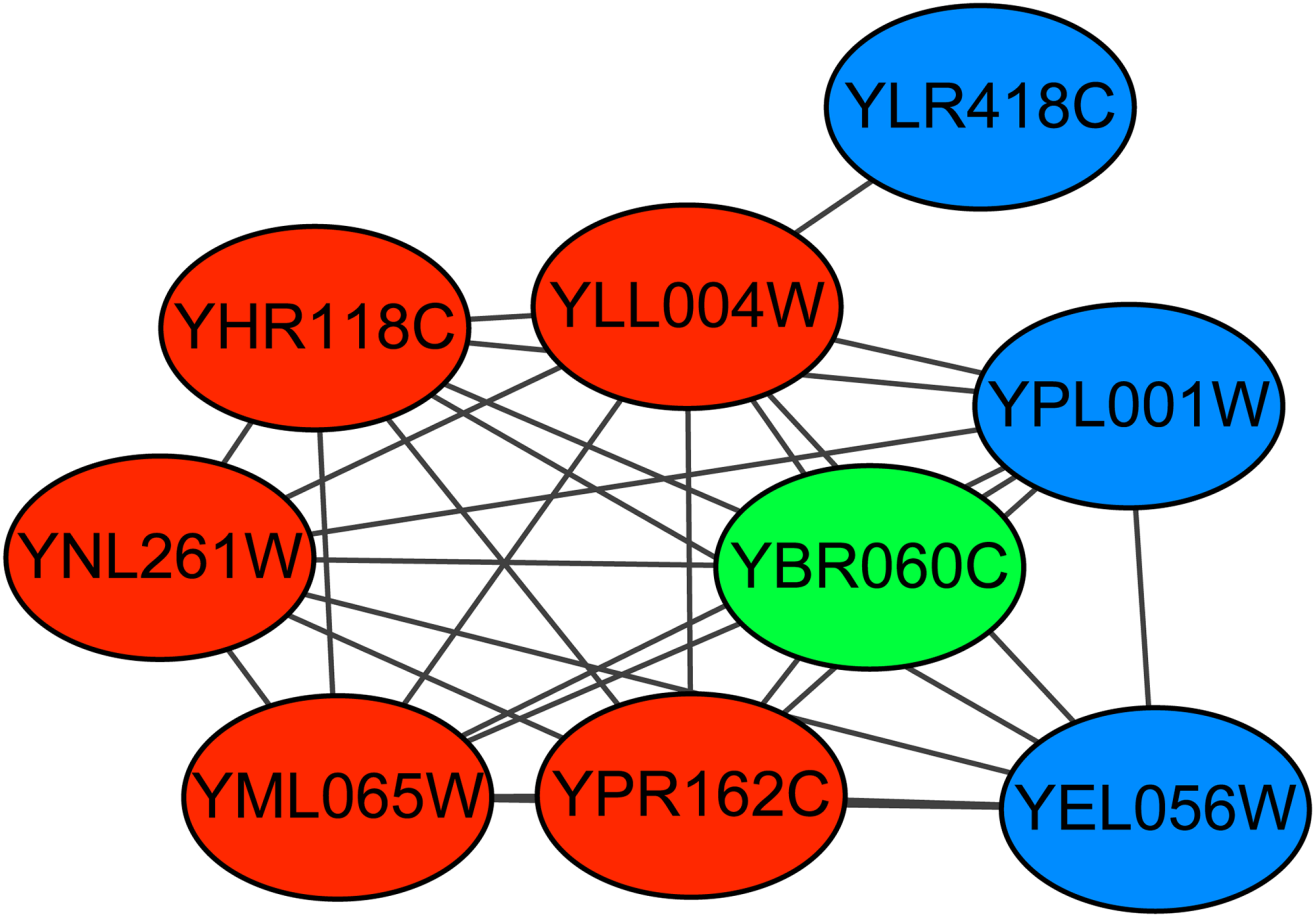
The retromer complex predicted by ClusterEPs. The red nodes represent the proteins in the true complex that are detected by the algorithm, the green nodes represent the proteins in the true complex that are not detected by the algorithm, and the blue nodes represent the proteins that do not belong to the true complex that are detected by the algorithm.

**Fig 8:**
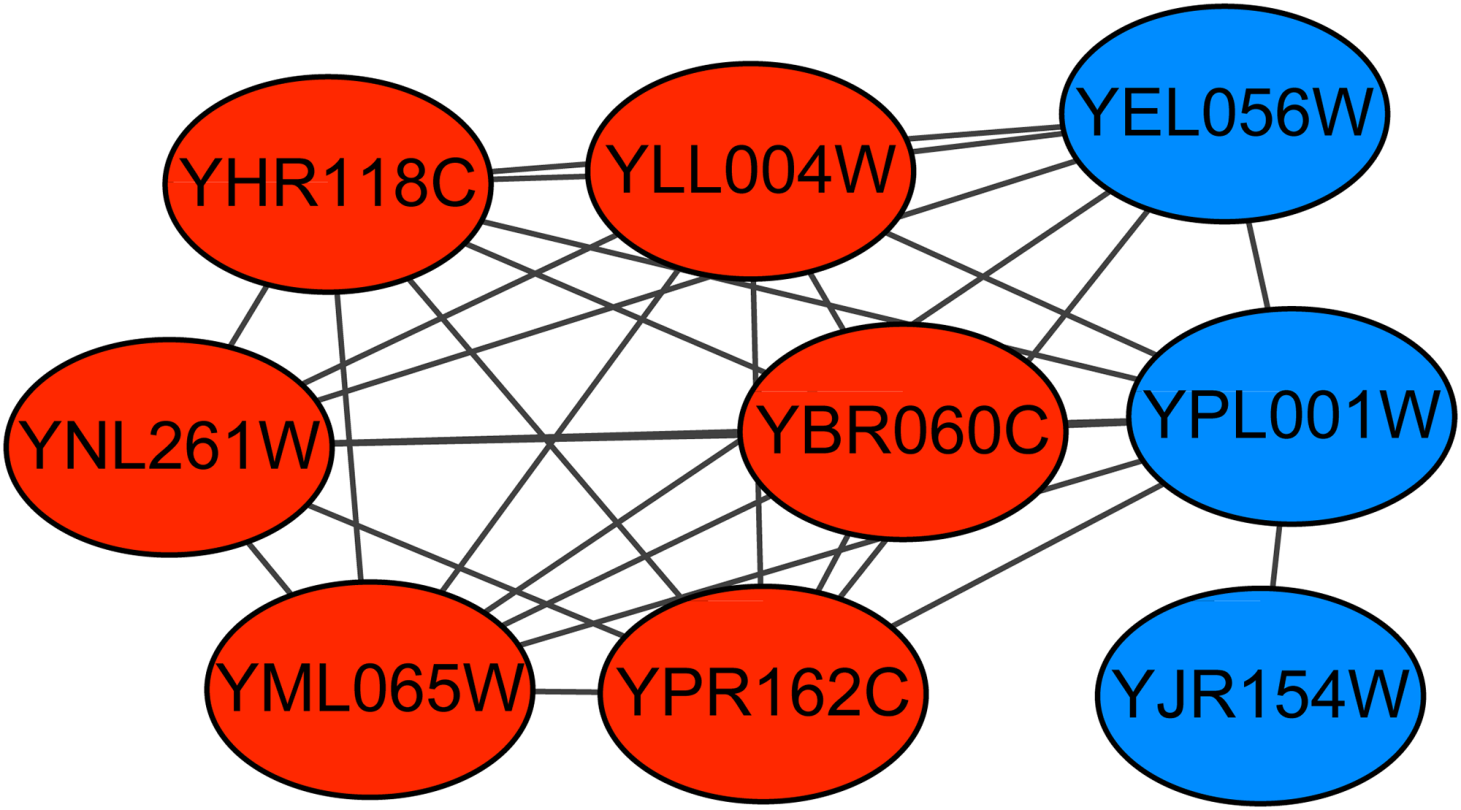
The retromer complex predicted by ClusterONE. The red nodes represent the proteins in the true complex that are detected by the algorithm, the green nodes represent the proteins in the true complex that are not detected by the algorithm, and the blue nodes represent the proteins that do not belong to the true complex that are detected by the algorithm.

The retromer complex is a central component for eukaryotic DNA replication, and it remains bound to chromatin at replication origins throughout the cell cycle [27] and contains 6 proteins. The Krogan extended PPI dataset contains the subgraph of this complex. Figs 6–8 show the predicted subgraphs of this complex by ClusterSS, ClusterEPs and ClusterONE, respectively. As shown, ClusterSS could detect the retromer complex completely and correctly. ClusterEPs missed one protein and added three unrelated proteins. Although ClusterONE found all the proteins of ORC, three unrelated proteins were added in the detection result.

**Fig 9:**
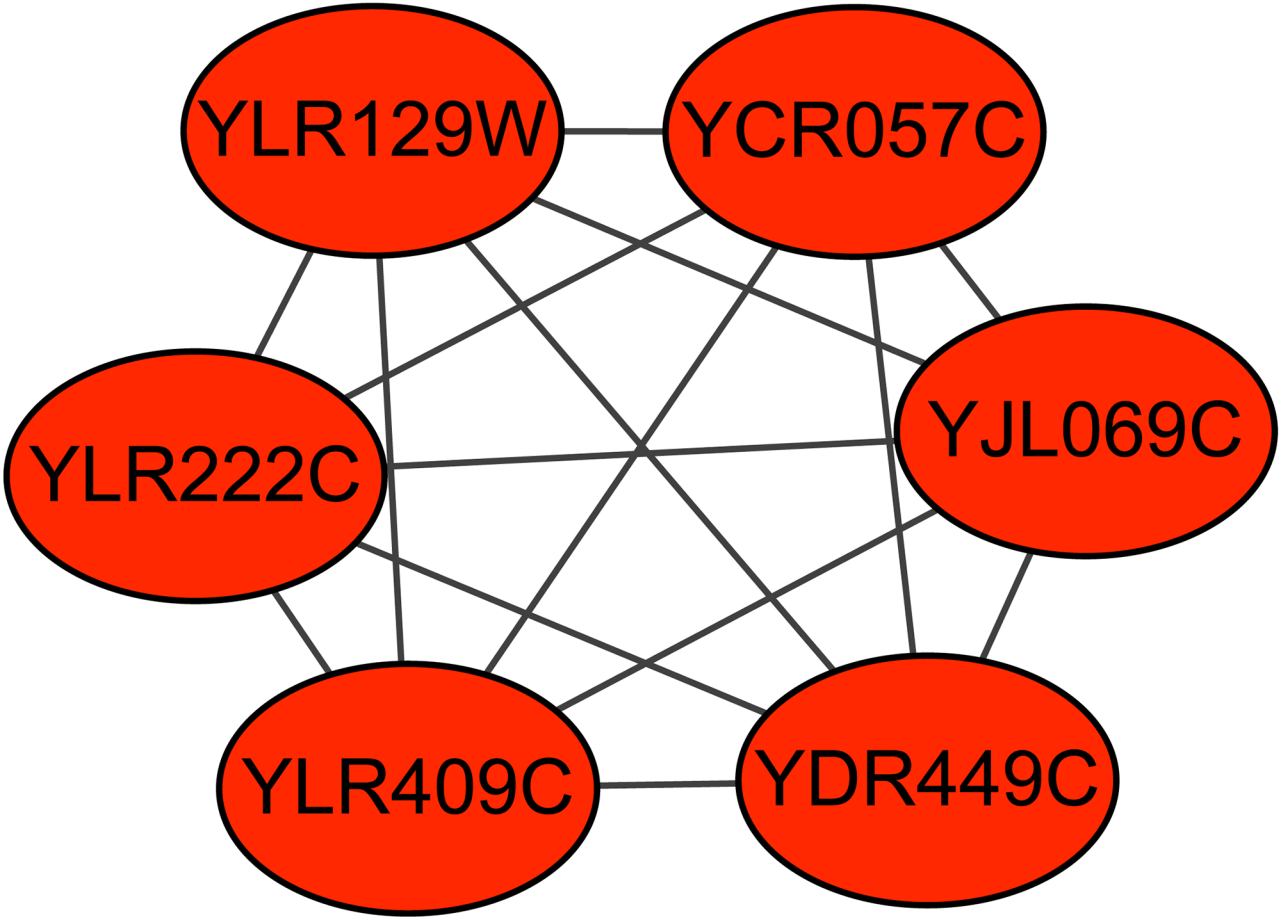
The Pwp2p-containing subcomplex of 90S preribosome predicted by ClusterSS. The red nodes represent the proteins in the true complex that are detected by the algorithm, the green nodes represent the proteins in the true complex that are not detected by the algorithm, and the blue nodes represent the proteins that do not belong to the true complex that are detected by the algorithm.

**Fig 10:**
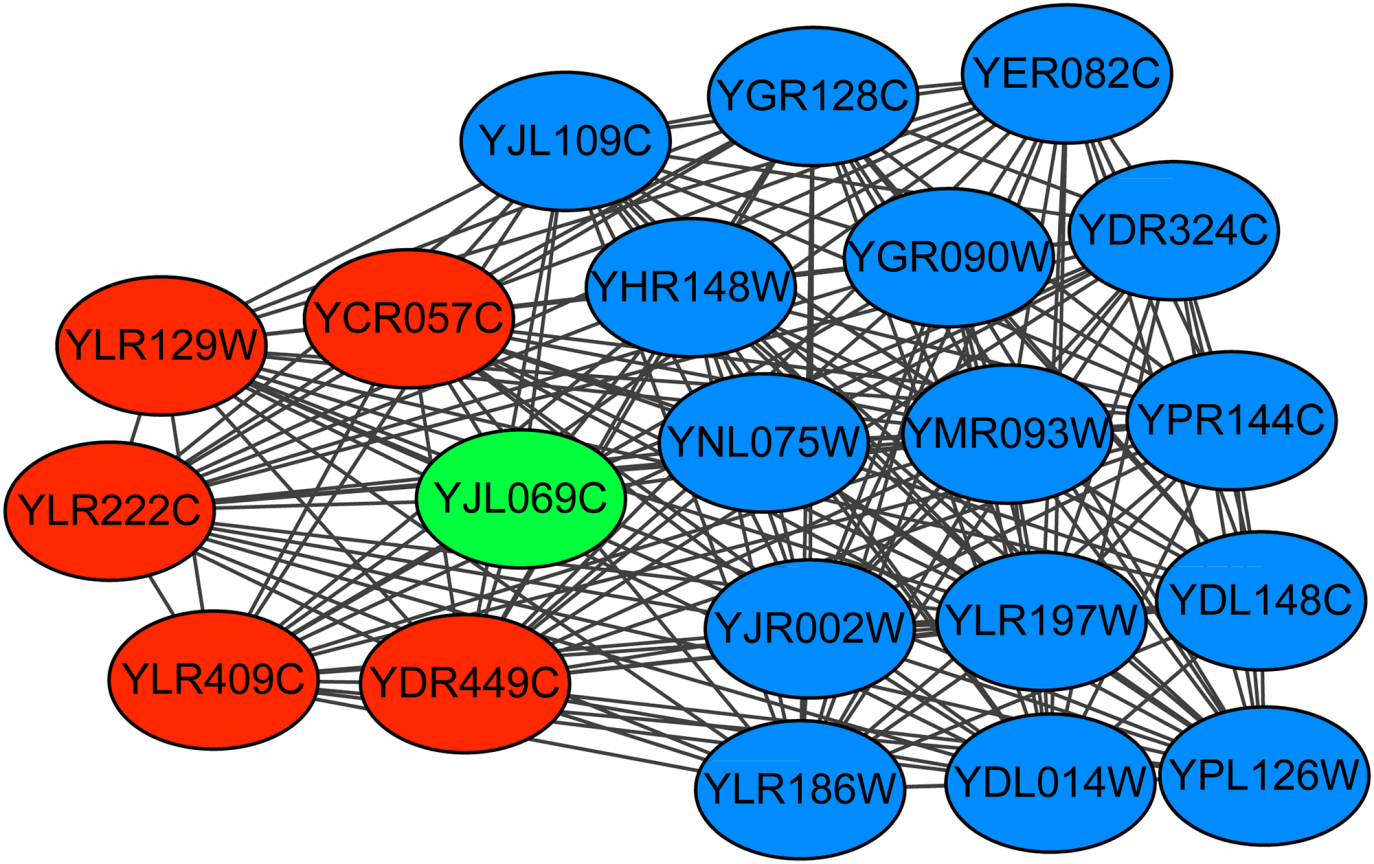
The Pwp2p-containing subcomplex of 90S preribosome predicted by ClusterEPs. The red nodes represent the proteins in the true complex that are detected by the algorithm, the green nodes represent the proteins in the true complex that are not detected by the algorithm, and the blue nodes represent the proteins that do not belong to the true complex that are detected by the algorithm.

**Fig 11:**
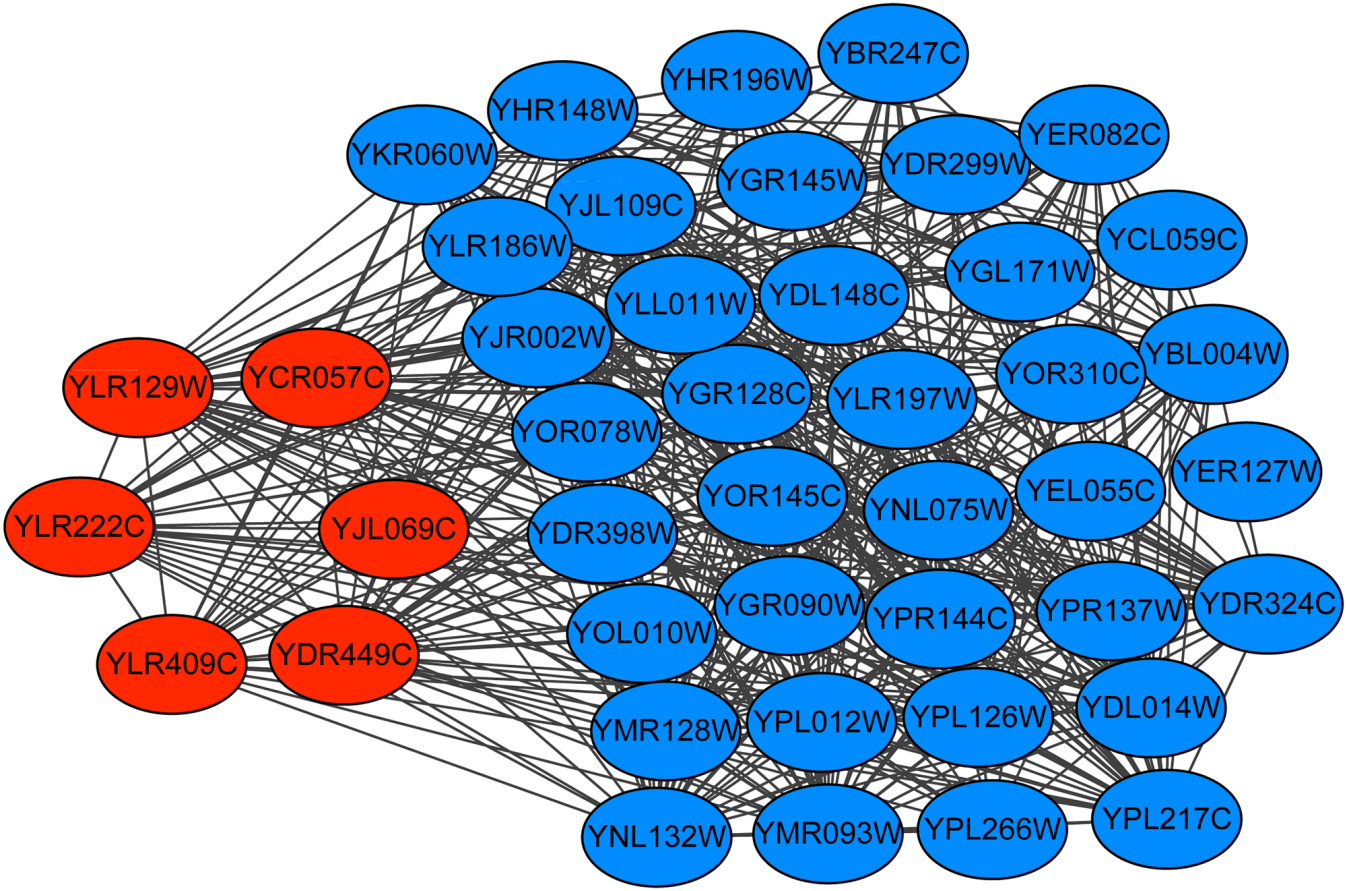
The Pwp2p-containing subcomplex of 90S preribosome predicted by ClusterONE. The red nodes represent the proteins in the true complex that are detected by the algorithm, the green nodes represent the proteins in the true complex that are not detected by the algorithm, and the blue nodes represent the proteins that do not belong to the true complex that are detected by the algorithm.

The Pwp2p-containing subcomplex of 90S preribosome contains 6 proteins, and the Collins PPI dataset contains the subgraph of this complex. Figs 9–11 show the predicted subgraphs of this complex by ClusterSS, ClusterEPs and ClusterONE, respectively. As shown, ClusterSS could detect the Pwp2p-containing subcomplex of 90S preribosome completely and correctly. ClusterEPs missed one protein and added fifteen unrelated proteins. Although ClusterONE found all the proteins of ORC, thirty-seven unrelated proteins were added in the detection result.

The DASH complex has been taken as a case study for ClusterONE and ClusterEPs, and the complex was embedded in the Krogan extended PPI dataset. Both ClusterONE and ClusterEPs can detect the complex correctly and clearly. ClusterSS can also detect this complex but adds an additional protein into the prediction result. The detail prediction results are shown in S1–S3 Figs.

The RSC and the SWI/SNF complexes were contained in the Collins PPI dataset and were also examined as a case study using ClusterONE and ClusterEPs. All three methods, ClusterONE, ClusterEPs and ClusterSS, can obtain prediction results close to the true complexes. The detailed detection results are shown in S4–S6 Figs.

### GO analysis of the new predicted complexes

Table 6 presents the GO analysis results of four complexes identified by ClusterSS. The match score represents the maximum overlapping score of predicted complexes with the MIPS and SGD complex datasets, which is calculated using Eq 6. The fourth column presents the minimum p-value of the matched GO terms, and the fifth column presents the corresponding descriptions.

**Table 6:** Four predicted complexes with low p-values that do not match the known complexes.

ID	complex	match score	min p-value	GO-Description
1	YNL308C YDL208W YHR072W-A YGL078C YCL059C YDL213C	0.0	4.22E-09	rRNA processing
2	YNL182C YDR101C YHR197W YER006W YCR072C YPR016C YGR245C YLR074C YER126C YPL093W YNR053C	0.0	3.11E-14	ribosome biogenesis
3	YDR235W YDL087C YPR057W YDR240C YLR298C YKL012W YBR119W YIL061C YLR275W YML046W YHR086W YGR013W	0.050	3.19E-25	small nuclear ribonucleoprotein complex
4	YDL030W YDL043C YAL032C YDR482C YMR240C YML049C YJL203W YPL151C YMR288W YOR319W	0.033	6.19E-20	spliceosomal complex

The first complex and the second complex are embedded in the Collins and Krogan extended PPI datasets, respectively, and they have no overlap with existing complexes in MIPS and SGD. The third complex and the fourth complex are embedded in the Gavin and Krogan core PPI datasets, respectively, and they have low overlapping scores with existing complexes. Table 6 presents the GO analysis results for the four predicted complexes obtained using BINGO [28]. All 6 proteins of complex-1 are enriched in 25 GO terms that are mostly related to rRNA processing, rRNA metabolic process or ncRNA processing (with a p-value < 6.88 * 10^−3^, and the minimum p-value is 4.22 * 10^−9^). All 11 proteins of complex-2 are enriched in 6 GO terms that are mostly related to ribosome biogenesis, ribonucleoprotein complex biogenesis or cellular component biogenesis (with a p-value < 5.25 * 10^−3^, and the minimum p-value is 3.11 * 10^−14^). All 12 proteins of complex-3 are enriched in 30 GO terms that are mostly related to small nuclear ribonucleoprotein complex, spliceosomal complex, RNA splicing or mRNA metabolic process (with a p-value < 7.43 * 10^−3^, and the minimum p-value is 3.19 * 10^−25^). All 10 proteins in complex-4 are enriched in 23 GO terms that are mostly related to spliceosomal complex, RNA splicing, mRNA processing or mRNA metabolic process (with a p-value < 8.47 * 10^−3^, and the minimum p-value is 6.19 * 10^−20^).

From the above GO analysis results, we observe that the proteins of each subgraph have close relationships according to the enriched GO terms. Although these subgraphs have not yet been characterized as complexes, they are very likely complexes in the biological sense. S8–S11 Tables provide detailed results of these GO enrichment analyses.

## Conclusion

The existing protein complex detection methods can be divided into two groups: unsupervised clustering methods and supervised search methods. Unsupervised clustering methods divide the PPI network into groups based on its topological structure, and most of these types of methods are sensitive to the density of the PPI network. The complexes that are too sparse or only a small part of highly dense subgraphs in the PPI network are difficult to detect using density-sensitive types of methods. The supervised search methods can learn a prediction model using the true complexes, but the noise in the PPI data and incomplete benchmark may cause the trained model to be inaccurate, which may misguide the protein complex search process.

In this paper, we provide a protein complex detection method that integrates these two types of methods by designing a score function that combines a classification model and structural information. We train a supervised neural network model on known protein complexes to obtain the supervised score, and we use a local structural score function to adjust the output of the neural network on each step of the protein complex search process. Based on the score function, we design a search method that works both forwards and backwards to detect the protein complexes. We conduct several comparative experiments on six benchmark PPI datasets and three complex datasets. Compared with the latest supervised method ClusterEPs, our method, ClusterSS, achieves a higher fraction score and composite score on all the PPI datasets under the same conditions. ClusterSS outperforms the semi-supervised method NN on the measures of precision, recall and F-measure. Compared with the unsupervised method, ClusterSS achieves the highest fraction, MMR and composite scores on all five PPI datasets when using SGD as the test set. Finally, we provide four examples of new predicted complexes, and the GO enrichment analysis shows that these complexes are very likely true complexes in the biological sense.

In future studies, we will integrate additional information, such as subcellular localization information and gene expression data, to make the classification model more accurate for detecting protein complexes.

## Supporting information

S1 Text Parameters of the neural network model. (PDF)

S1 Table The feature used to describing subgraph. (PDF)

S2 Table The composite score of ClusterSS with different values of alpha using SGD as the test set. (PDF)

S3 Table The composite score of ClusterSS with different values of alpha using MIPS as the test set. (PDF)

S4 Table The cluster number of ClusterSS with different values of alpha using SGD as the test set. (PDF)

S5 Table The cluster number of ClusterSS with different values of alpha using MIPS as the test set. (PDF)

S6 Table The running time of ClusterSS. (PDF)

S7 Table The performance of fast and slow versions of ClusterSS. (PDF)

S8 Table GO functional enrichment analysis for complex-1. (PDF)

S9 Table GO functional enrichment analysis for complex-2. (PDF)

S10 Table The GO functional enrichment analysis for complex-3. (PDF)

S11 Table The GO functional enrichment analysis for complex-4. (PDF)

S1 Fig The DASH complex predicted by ClusterSS. (PDF)

S2 Fig The DASH complex predicted by ClusterEPs. (PDF)

S3 Fig The DASH complex predicted by ClusterONE. (PDF)

S4 Fig The RSC and SWI/SNF complexes predicted by ClusterSS. (PDF)

S5 Fig The RSC and SWI/SNF complexes predicted by ClusterEPs. (PDF)

S6 Fig The RSC and SWI/SNF complexes predicted by ClusterONE. (PDF)

S1 Dataset The DIP PPI dataset. (TXT)

S2 Dataset The Gavin PPI dataset. (TXT)

S3 Dataset The Krogan core PPI dataset. (TXT)

S4 Dataset The Krogan extended PPI dataset. (TXT)

S5 Dataset The BioGRID PPI dataset. (TXT)

S6 Dataset The Collins PPI dataset. (TXT)

S7 Dataset The TAP06 protein complex dataset. (TXT)

S8 Dataset The MIPS protein complex dataset. (TXT)

S9 Dataset The SGD protein complex dataset. (TXT)
