# Supplementary material for: Predicting protein complexes using a supervised learning method combined with local structural information
Source: PLoS One. 2018 Mar 19;13(3):e0194124. doi: 10.1371/journal.pone.0194124 (PMC5858846; doi:10.1371/journal.pone.0194124)
Supplement: S1 Text — (PDF) [file pone.0194124.s001.pdf]

Parameters of the neural network model

In this section, we presented the detailed parameters of the neural network model we used.

1) number of epochs: 500

2) learning rate: 0.3

3) momentum: 0.2

4) squash function: sigmoid function

5) loss function: least square function

6) initial weights: random value range from -0.05 to 0.05
