# Supplementary material for: Predicting protein complexes using a supervised learning method combined with local structural information
Source: PLoS One. 2018 Mar 19;13(3):e0194124. doi: 10.1371/journal.pone.0194124 (PMC5858846; doi:10.1371/journal.pone.0194124)
Supplement: S1 Table — (PDF) [file pone.0194124.s002.pdf]

S1 Table: The feature used to describing subgraph.

group name	#features	description
Node Size	1	The number of node in the subgraph.
Graph Density	1	The density of the subgraph.
Degree statistics	4	The mean, variance, median and maximum of the degrees of the nodes in the subgraph.
Edge Weight	2	The mean and variance of the weights of edges in the subgraph.
Degree Correlation Statistics	3	The mean, variance and maximum of the degree correlation
Clustering Coefficient Statistics	3	The mean, variance and maximum of the clustering coefficient
Topological Coefficients	3	The mean, variance and maximum of the topological coefficient
First Eigenvalues	3	The first three largest singular values of the subgraph's adjacency matrix.
Protein Weight/Size	4	The mean and maximum of protein length and the mean and maximum protein weights.
