# Supplementary material for: Predicting protein complexes using a supervised learning method combined with local structural information
Source: PLoS One. 2018 Mar 19;13(3):e0194124. doi: 10.1371/journal.pone.0194124 (PMC5858846; doi:10.1371/journal.pone.0194124)
Supplement: S2 Table — (PDF) [file pone.0194124.s003.pdf]

S2 Table: The composite score of ClusterSS with different values of alpha using SGD as the test set.

Dataset \ α	1	1.01	1.02	1.03	1.04	1.05	1.1	1.2	1.3
Gavin	1.914	1.963	**2.028**	2.017	2.027	1.923	1.725	1.443	1.419
Krogan core	1.844	1.884	**1.946**	1.921	1.892	1.782	1.617	1.439	1.359
Krogan extended	1.652	1.721	**1.784**	1.777	1.698	1.616	1.450	1.291	1.228
Collins	2.113	2.158	**2.211**	2.139	2.021	1.942	1.736	1.514	1.438
BioGRID	1.717	**1.756**	1.559	1.287	1.182	1.054	0.969	0.915	0.835
