# Supplementary material for: Predicting protein complexes using a supervised learning method combined with local structural information
Source: PLoS One. 2018 Mar 19;13(3):e0194124. doi: 10.1371/journal.pone.0194124 (PMC5858846; doi:10.1371/journal.pone.0194124)
Supplement: S3 Table — (PDF) [file pone.0194124.s004.pdf]

S3 Table: The composite score of ClusterSS with different values of alpha using MIPS as the test set.

Dataset \ α	1	1.01	1.02	1.03	1.04	1.05	1.1	1.2	1.3
Gavin	1.441	1.505	**1.593**	1.515	1.541	1.472	1.311	1.085	1.045
Krogan core	1.463	1.499	**1.536**	1.504	1.440	1.378	1.227	1.082	1.005
Krogan extended	1.312	1.382	**1.429**	1.417	1.340	1.315	1.133	0.971	0.921
Collins	1.684	1.681	**1.732**	1.633	1.542	1.481	1.325	1.124	1.036
BioGRID	**1.364**	1.335	1.125	0.971	0.880	0.802	0.771	0.729	0.666
