# Supplementary material for: Predicting protein complexes using a supervised learning method combined with local structural information
Source: PLoS One. 2018 Mar 19;13(3):e0194124. doi: 10.1371/journal.pone.0194124 (PMC5858846; doi:10.1371/journal.pone.0194124)
Supplement: S4 Table — (PDF) [file pone.0194124.s005.pdf]

S4 Table: The cluster number of ClusterSS with different values of alpha using SGD as the test set.

Dataset \ α	1	1.01	1.02	1.03	1.04	1.05	1.1	1.2	1.3
Gavin	343	393	478	583	664	724	875	1011	1013
Krogan core	633	696	762	861	977	1071	1259	1409	1434
Krogan extended	756	794	925	1064	1210	1290	1501	1596	1633
Collins	258	377	473	613	709	745	877	991	1023
BioGRID	3260	3918	4323	4531	4678	4755	4920	5015	5056
