# Supplementary material for: Predicting protein complexes using a supervised learning method combined with local structural information
Source: PLoS One. 2018 Mar 19;13(3):e0194124. doi: 10.1371/journal.pone.0194124 (PMC5858846; doi:10.1371/journal.pone.0194124)
Supplement: S5 Table — (PDF) [file pone.0194124.s006.pdf]

S5 Table: The cluster number of ClusterSS with different values of alpha using MIPS as the test set.

Dataset \ α	1	1.01	1.02	1.03	1.04	1.05	1.1	1.2	1.3
Gavin	326	373	474	584	651	727	879	1025	1044
Krogan core	611	654	749	856	957	1052	1237	1380	1429
Krogan extended	733	785	909	1055	1197	1282	1485	1611	1632
Collins	247	372	467	609	699	738	869	989	1022
BioGRID	3162	3831	4032	4385	4524	4575	4708	4866	4902
