# Supplementary material for: Predicting protein complexes using a supervised learning method combined with local structural information
Source: PLoS One. 2018 Mar 19;13(3):e0194124. doi: 10.1371/journal.pone.0194124 (PMC5858846; doi:10.1371/journal.pone.0194124)
Supplement: S6 Table — (PDF) [file pone.0194124.s007.pdf]

S6 Table: **The running time of ClusterSS.** 'N/A' represents the algorithm can not give any results within 24 hours

| Dataset | Test set | slow ClusterSS (s) | fast ClusterSS (s) |
|---|---|---|---|
| Collins | SGD | 3547.34 | 2360.47 |
| | MIPS | 3594.90 | 2920.47 |
| Krogan core | SGD | 3309.37 | 2383.02 |
| | MIPS | 3223.21 | 2882.98 |
| Krogan extended | SGD | 4097.73 | 2432.19 |
| | MIPS | 4174.99 | 2845.42 |
| Gavin | SGD | 3381.93 | 2339.74 |
| | MIPS | 3377.22 | 2899.51 |
| BioGRID | SGD | N/A | 7545.00 |
| | MIPS | N/A | 9770.17 |
