# Supplementary material for: Predicting protein complexes using a supervised learning method combined with local structural information
Source: PLoS One. 2018 Mar 19;13(3):e0194124. doi: 10.1371/journal.pone.0194124 (PMC5858846; doi:10.1371/journal.pone.0194124)
Supplement: S7 Table — (PDF) [file pone.0194124.s008.pdf]

S7 Table: **The performance of fast and slow versions of ClusterSS.** 'N/A' represents the algorithm can not give any results within 24 hours

Dataset	Test set	Method	Frac	Acc	MMR	Composite score
Collins	SGD	fast ClusterSS	0.885	0.737	0.588	2.211
	SGD	slow ClusterSS	0.866	0.729	0.578	2.172
	MIPS	fast ClusterSS	0.773	0.521	0.438	1.732
	MIPS	slow ClusterSS	0.773	0.507	0.435	1.715
Krogan core	SGD	fast ClusterSS	0.788	0.654	0.504	1.946
	SGD	slow ClusterSS	0.776	0.651	0.484	1.911
	MIPS	fast ClusterSS	0.704	0.449	0.382	1.536
	MIPS	slow ClusterSS	0.717	0.448	0.370	1.536
Krogan extended	SGD	fast ClusterSS	0.720	0.614	0.450	1.784
	SGD	slow ClusterSS	0.706	0.605	0.434	1.745
	MIPS	fast ClusterSS	0.647	0.428	0.354	1.429
	MIPS	slow ClusterSS	0.652	0.422	0.352	1.426
Gavin	SGD	fast ClusterSS	0.808	0.715	0.504	2.028
	SGD	slow ClusterSS	0.806	0.705	0.501	2.012
	MIPS	fast ClusterSS	0.732	0.485	0.376	1.593
	MIPS	slow ClusterSS	0.674	0.484	0.360	1.518
BioGRID	SGD	fast ClusterSS	0.639	0.531	0.390	1.559
	SGD	slow ClusterSS	N/A	N/A	N/A	N/A
	MIPS	fast ClusterSS	0.514	0.336	0.275	1.125
	MIPS	slow ClusterSS	N/A	N/A	N/A	N/A
