# Supplementary material for: Predicting protein complexes using a supervised learning method combined with local structural information
Source: PLoS One. 2018 Mar 19;13(3):e0194124. doi: 10.1371/journal.pone.0194124 (PMC5858846; doi:10.1371/journal.pone.0194124)
Supplement: S8 Table — (PDF) [file pone.0194124.s009.pdf]

S8 Table: GO functional enrichment analysis for complex-1

6364	rRNA processing	4.22E-09	all 6 genes
16072	rRNA metabolic process	5.35E-09	all 6 genes
5730	nucleolus	6.56E-09	all 6 genes
34470	ncRNA processing	3.08E-08	all 6 genes
42254	ribosome biogenesis	4.46E-08	all 6 genes
34660	ncRNA metabolic process	7.90E-08	all 6 genes
22613	ribonucleoprotein complex biogenesis	9.68E-08	all 6 genes
6396	RNA processing	3.86E-07	all 6 genes
31981	nuclear lumen	4.51E-07	all 6 genes
70013	intracellular organelle lumen	4.02E-06	all 6 genes
43233	organelle lumen	4.02E-06	all 6 genes
31974	membrane-enclosed lumen	5.58E-06	all 6 genes
16070	RNA metabolic process	5.91E-06	all 6 genes
44085	cellular component biogenesis	9.34E-06	all 6 genes
43228	non-membrane-bounded organelle	2.71E-05	all 6 genes
43232	intracellular non-membrane-bounded organelle	2.71E-05	all 6 genes
44428	nuclear part	3.81E-05	all 6 genes
90304	nucleic acid metabolic process	1.67E-04	all 6 genes
6139	nucleobase, nucleoside, nucleotide and nucleic acid metabolic process	4.07E-04	all 6 genes
10467	gene expression	8.73E-04	all 6 genes
34641	cellular nitrogen compound metabolic process	8.76E-04	all 6 genes
6807	nitrogen compound metabolic process	1.02E-03	all 6 genes
5634	nucleus	1.58E-03	all 6 genes
44422	organelle part	6.88E-03	all 6 genes
44446	intracellular organelle part	6.88E-03	all 6 genes
