# Supplementary material for: Predicting protein complexes using a supervised learning method combined with local structural information
Source: PLoS One. 2018 Mar 19;13(3):e0194124. doi: 10.1371/journal.pone.0194124 (PMC5858846; doi:10.1371/journal.pone.0194124)
Supplement: S9 Table — (PDF) [file pone.0194124.s010.pdf]

S9 Table: GO functional enrichment analysis for complex-2

42254	ribosome biogenesis	3.11E-14	all 11 genes
22613	ribonucleoprotein complex biogenesis	1.30E-13	all 11 genes
44085	cellular component biogenesis	5.85E-10	all 11 genes
5634	nucleus	7.28E-06	all 11 genes
43227	membrane-bounded organelle	5.25E-03	all 11 genes
43231	intracellular membrane-bounded organelle	5.25E-03	all 11 genes
