# Supplementary material for: Predicting protein complexes using a supervised learning method combined with local structural information
Source: PLoS One. 2018 Mar 19;13(3):e0194124. doi: 10.1371/journal.pone.0194124 (PMC5858846; doi:10.1371/journal.pone.0194124)
Supplement: S10 Table — (PDF) [file pone.0194124.s011.pdf]

S10 Table: GO functional enrichment analysis for complex-3

GO ID	Description	p-value	genes
30532	small nuclear ribonucleoprotein complex	3.19E-25	all 12 genes
5681	spliceosomal complex	7.57E-24	all 12 genes
398	nuclear mRNA splicing, via spliceosome	2.26E-22	all 12 genes
377	RNA splicing, via transesterification reactions with bulged adenosine as nucleophile	2.55E-22	all 12 genes
375	RNA splicing, via transesterification reactions	6.53E-22	all 12 genes
8380	RNA splicing	1.53E-20	all 12 genes
6397	mRNA processing	1.55E-18	all 12 genes
16071	mRNA metabolic process	4.52E-17	all 12 genes
6396	RNA processing	1.40E-13	all 12 genes
30529	ribonucleoprotein complex	2.39E-13	all 12 genes
16070	RNA metabolic process	3.37E-11	all 12 genes
44428	nuclear part	1.42E-09	all 12 genes
90304	nucleic acid metabolic process	2.72E-08	all 12 genes
6139	nucleobase, nucleoside, nucleotide and nucleic acid metabolic process	1.63E-07	all 12 genes
32991	macromolecular complex	4.17E-07	all 12 genes
10467	gene expression	7.53E-07	all 12 genes
34641	cellular nitrogen compound metabolic process	7.57E-07	all 12 genes
6807	nitrogen compound metabolic process	1.03E-06	all 12 genes
5634	nucleus	2.48E-06	all 12 genes
44446	intracellular organelle part	4.70E-05	all 12 genes
44422	organelle part	4.70E-05	all 12 genes
44260	cellular macromolecule metabolic process	1.68E-04	all 12 genes
43170	macromolecule metabolic process	2.27E-04	all 12 genes
44238	primary metabolic process	1.98E-03	all 12 genes
44237	cellular metabolic process	2.99E-03	all 12 genes
43231	intracellular membrane-bounded organelle	3.26E-03	all 12 genes
43227	membrane-bounded organelle	3.26E-03	all 12 genes
8152	metabolic process	5.23E-03	all 12 genes
43229	intracellular organelle	7.43E-03	all 12 genes
43226	organelle	7.43E-03	all 12 genes
