# Supplementary material for: Predicting protein complexes using a supervised learning method combined with local structural information
Source: PLoS One. 2018 Mar 19;13(3):e0194124. doi: 10.1371/journal.pone.0194124 (PMC5858846; doi:10.1371/journal.pone.0194124)
Supplement: S11 Table — (PDF) [file pone.0194124.s012.pdf]

S11 Table: GO functional enrichment analysis for complex-4

GO ID	Description	p-value	genes
5681	spliceosomal complex	6.19E-20	all 10 genes
8380	RNA splicing	3.30E-17	all 10 genes
6397	mRNA processing	1.51E-15	all 10 genes
16071	mRNA metabolic process	2.48E-14	all 10 genes
6396	RNA processing	1.98E-11	all 10 genes
30529	ribonucleoprotein complex	3.08E-11	all 10 genes
16070	RNA metabolic process	1.89E-09	all 10 genes
44428	nuclear part	4.26E-08	all 10 genes
90304	nucleic acid metabolic process	4.99E-07	all 10 genes
6139	nucleobase, nucleoside, nucleotide and nucleic acid metabolic process	2.22E-06	all 10 genes
32991	macromolecular complex	4.84E-06	all 10 genes
10467	gene expression	7.92E-06	all 10 genes
34641	cellular nitrogen compound metabolic process	7.96E-06	all 10 genes
6807	nitrogen compound metabolic process	1.03E-05	all 10 genes
5634	nucleus	2.14E-05	all 10 genes
44422	organelle part	2.48E-04	all 10 genes
44446	intracellular organelle part	2.48E-04	all 10 genes
44260	cellular macromolecule metabolic process	7.17E-04	all 10 genes
43170	macromolecule metabolic process	9.21E-04	all 10 genes
44238	primary metabolic process	5.60E-03	all 10 genes
44237	cellular metabolic process	7.89E-03	all 10 genes
43231	intracellular membrane-bounded organelle	8.47E-03	all 10 genes
43227	membrane-bounded organelle	8.47E-03	all 10 genes
