# Supplementary material for: Predicting protein complexes using a supervised learning method combined with local structural information
Source: PLoS One. 2018 Mar 19;13(3):e0194124. doi: 10.1371/journal.pone.0194124 (PMC5858846; doi:10.1371/journal.pone.0194124)

S1 Fig: The DASH complex predicted by ClusterSS. The red nodes represent the proteins in the true complex that are detected by the algorithm, and the blue nodes represent the proteins that do not belong to the true complex that are detected by the algorithm.

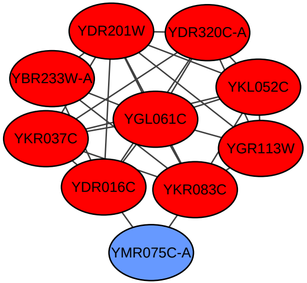

Supplement: S1 Fig — (PDF) [file pone.0194124.s013.pdf]
