# Supplementary material for: Predicting protein complexes using a supervised learning method combined with local structural information
Source: PLoS One. 2018 Mar 19;13(3):e0194124. doi: 10.1371/journal.pone.0194124 (PMC5858846; doi:10.1371/journal.pone.0194124)

S4 Fig: The RSC and SWI/SNF complexes predicted by ClusterSS. The red and green nodes represent the proteins belong to RSC and SWI/SNF complex, respectively; the yellow nodes represent the proteins belong to both complexes, while the blue nodes belong to neither. The shade areas represent the complexes predicted by the algorithm.

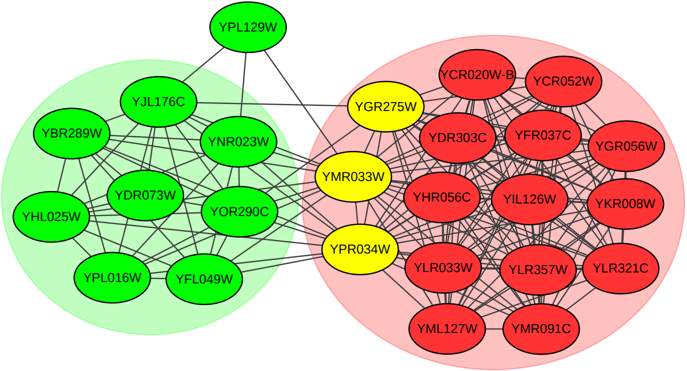

Supplement: S4 Fig — (PDF) [file pone.0194124.s016.pdf]
